# Diamond formation kinetics in shock-compressed C─H─O samples recorded by small-angle x-ray scattering and x-ray diffraction

**DOI:** 10.1126/sciadv.abo0617

**Published:** 2022-09-02

**Authors:** Zhiyu He, Melanie Rödel, Julian Lütgert, Armin Bergermann, Mandy Bethkenhagen, Deniza Chekrygina, Thomas E. Cowan, Adrien Descamps, Martin French, Eric Galtier, Arianna E. Gleason, Griffin D. Glenn, Siegfried H. Glenzer, Yuichi Inubushi, Nicholas J. Hartley, Jean-Alexis Hernandez, Benjamin Heuser, Oliver S. Humphries, Nobuki Kamimura, Kento Katagiri, Dimitri Khaghani, Hae Ja Lee, Emma E. McBride, Kohei Miyanishi, Bob Nagler, Benjamin Ofori-Okai, Norimasa Ozaki, Silvia Pandolfi, Chongbing Qu, Divyanshu Ranjan, Ronald Redmer, Christopher Schoenwaelder, Anja K. Schuster, Michael G. Stevenson, Keiichi Sueda, Tadashi Togashi, Tommaso Vinci, Katja Voigt, Jan Vorberger, Makina Yabashi, Toshinori Yabuuchi, Lisa M. V. Zinta, Alessandra Ravasio, Dominik Kraus

**Affiliations:** ^1^ Helmholtz-Zentrum Dresden-Rossendorf, Bautzner Landstrasse 400, 01328 Dresden, Germany.; ^2^ Institut für Physik, Universität Rostock, Albert-Einstein-Str. 23-24, 18059 Rostock, Germany.; ^3^ Shanghai Institute of Laser Plasma, 201800 Shanghai, China.; ^4^ Technische Universität Dresden, 01069 Dresden, Germany.; ^5^ École Normale Supérieure de Lyon, Laboratoire de Géologie de Lyon, LGLTPE UMR 5276, Centre Blaise Pascal, 46 allée d’Italie, Lyon 69364, France.; ^6^ SLAC National Accelerator Laboratory, Menlo Park, CA 94025, USA.; ^7^ Stanford University, Stanford, CA 94305, USA.; ^8^ Japan Synchrotron Radiation Research Institute, 1-1-1 Kouto, Sayo-cho, Sayo-gun, Hyogo 679-5198, Japan.; ^9^ RIKEN SPring-8 Center, 1-1-1 Kouto, Sayo-cho, Sayo-gun, Hyogo 679-5148, Japan.; ^10^ Centre for Earth Evolution and Dynamics, University of Oslo, N-0315 Oslo, Norway.; ^11^ European Synchrotron Radiation Facility, 71 avenue des Martyrs, 38000 Grenoble, France.; ^12^ Graduate School of Engineering, Osaka University, Suita, Osaka 565-0871, Japan.; ^13^ Institute of Laser Engineering, Osaka University, Suita, Osaka 565-0871, Japan.; ^14^ Erlangen Centre for Astroparticle Physics, Friedrich-Alexander-Universität Erlangen Nürnberg, Erwin-Rommel-Str 1, 91058 Erlangen, Germany.; ^15^ LULI, CNRS, CEA, Sorbonne Université, Ecole Polytechnique–Institut Polytechnique de Paris, F-91128 Palaiseau, France.

## Abstract

Extreme conditions inside ice giants such as Uranus and Neptune can result in peculiar chemistry and structural transitions, e.g., the precipitation of diamonds or superionic water, as so far experimentally observed only for pure C─H and H_2_O systems, respectively. Here, we investigate a stoichiometric mixture of C and H_2_O by shock-compressing polyethylene terephthalate (PET) plastics and performing in situ x-ray probing. We observe diamond formation at pressures between 72 ± 7 and 125 ± 13 GPa at temperatures ranging from ~3500 to ~6000 K. Combining x-ray diffraction and small-angle x-ray scattering, we access the kinetics of this exotic reaction. The observed demixing of C and H_2_O suggests that diamond precipitation inside the ice giants is enhanced by oxygen, which can lead to isolated water and thus the formation of superionic structures relevant to the planets’ magnetic fields. Moreover, our measurements indicate a way of producing nanodiamonds by simple laser-driven shock compression of cheap PET plastics.

## Teaser

Laser compression of PET plastics mimics the chemistry inside Uranus and may offer a way to simply produce nanodiamonds.

## INTRODUCTION

Ice giant planets such as Neptune and Uranus are highly abundant in our galaxy (*1*). The interiors of these celestial objects are thought to be mainly composed of a dense fluid mixture of water, methane, and ammonia (*2*). Because of the high pressures and temperatures deep inside these planets, the material mixture will likely undergo chemical reactions and structural transitions (*3*–*5*). An example of these reactions is the possible dissociation of hydrocarbons (*6*) and subsequent phase separation, allowing the formation of diamonds (*7*) and presumably metallic hydrogen or superionic water (*3*, *8*), which may act as a heat source and help to explain the generation of the unique magnetic fields modeled for the ice giants (*9*–*11*).

Recent laser shock experiments on polystyrene [PS; (C_8_H_8_)*_n_*] in combination with x-ray techniques have provided the first in situ evidence for the formation of diamonds in compressed hydrocarbons at planetary-relevant states in the laboratory (*12*–*14*). However, the presence of water and therefore large amounts of oxygen needs to be considered for further conclusions on processes inside the ice giants. Thus, investigating C─H─O samples provides a more realistic scenario than studying pure hydrocarbon or water systems (*15*). A separation of carbon from H_2_O is likely required for a superionic phase of water to be present inside Neptune and/or Uranus (*3*). If carbon would form bonds with oxygen, such structures, which could help to explain the peculiar magnetic field observed for both planets, may be inhibited.

Thanks to the advent of x-ray free-electron laser (XFEL) facilities and increasingly mature experimental capabilities, probing the internal structure of materials under dynamic compression to mimic planetary interiors has seen tremendous progress in recent years. While x-ray diffraction (XRD) identifies crystalline and liquid correlations on the angstrom level, small-angle x-ray scattering (SAXS) (*16*) is sensitive to feature sizes on the order of 1 to 100 nm. Therefore, combining SAXS and XRD in a single experiment has a great potential to enable accurate measurements of the nanodiamond size distribution and nucleation process initiated inside the plastic samples, which provides direct access to the kinetics of the carbon-hydrogen phase separation reaction relevant to the interiors of planets (*13*).

Here, the size distribution and the growth process of nanodiamonds, created from shock-compressed polyethylene terephthalate [PET; (C_10_H_8_O_4_)*_n_*, stoichiometrically a mixture of carbon and H_2_O], are characterized by in situ XRD and SAXS consistently at two different XFEL facilities, which shows the importance of the pressure *P*–temperature *T* state on the diamond formation kinetics. Standard velocity interferometer for any reflector (VISAR) diagnostics and the recently published equation of state data for PET (*15*) are applied to estimate the *P*-*T* state of the shocked PET sample, allowing us to compare the XRD data to density functional theory molecular dynamics (DFT-MD) simulations, where they show excellent agreement. Our results provide insights of unprecedented quality into chemistry relevant to planetary interiors and the general capabilities for simultaneously characterizing structural transitions on both angstrom and nanometer scale in dynamic compression experiments. At the same time, as diamond formation is achieved by a single-shock compression in contrast to more elaborate compression histories required in previous experiments (*12*), our study points toward a new way to efficiently produce nanodiamonds using cheap PET plastics as initial material.

## RESULTS

### Experimental method

**Fig. 1.**
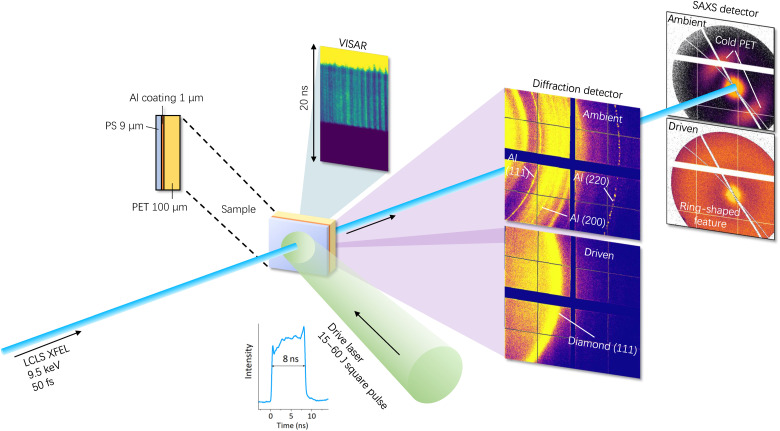
Schematic of the experimental setup combining XRD, SAXS, and VISAR at the MEC endstation of LCLS. Raw XRD and SAXS data under ambient and driven conditions of PET are presented. The SAXS is centered around the direct XFEL beam, with high-Z wires used to block the zero momentum-transfer peak. Images under ambient and driven conditions apply the same color scale.

We performed the discussed experiments at two different XFEL facilities: the Matter in Extreme Conditions (MEC) endstation of the Linac Coherent Light Source (LCLS) of SLAC National Accelerator Laboratory (*17*, *18*) (Fig. 1) and the SPring-8 Angstrom Compact free electron LAser (SACLA) (*19*, *20*). At LCLS, the structural changes and density variations of compressed PET can be observed by in situ XRD and SAXS with the LCLS pulse of 9.5-keV photon energy and 50-fs duration. The XRD and SAXS detectors were capable of recording single-photon events, and the data were integrated azimuthally after masking the beamstop and the parasitic scattering. More details on the experimental setup and the applied diagnostics can be found in Materials and Methods. A similar setup was used at SACLA and is depicted in fig. S1 of the Supplementary Materials.

Examples of raw data images from XRD and SAXS from PET under ambient conditions and a shocked state are illustrated in Fig. 1. Shocked and ambient data apply the same color scale per diagnostic. The drive laser ablates the aluminum coating that is visible in the XRD lineouts of cold PET samples. Hence, these peaks disappear for driven targets (see Fig. 1). In the shocked state, the symmetric scattering characteristic of ambient PET in the SAXS image vanishes. In turn, the SAXS signal increases strongly in the higher–wave number *q* range of the detector and results in a bright ring-shaped feature, which corresponds to the pattern consistent with densely packed nanospheres (*21*). Contemporaneously, strong diamond diffraction is observed by XRD, which demonstrates the formation of nanodiamonds being traceable by two complementary x-ray techniques.

### Diamond formation kinetics

**Fig. 2.**
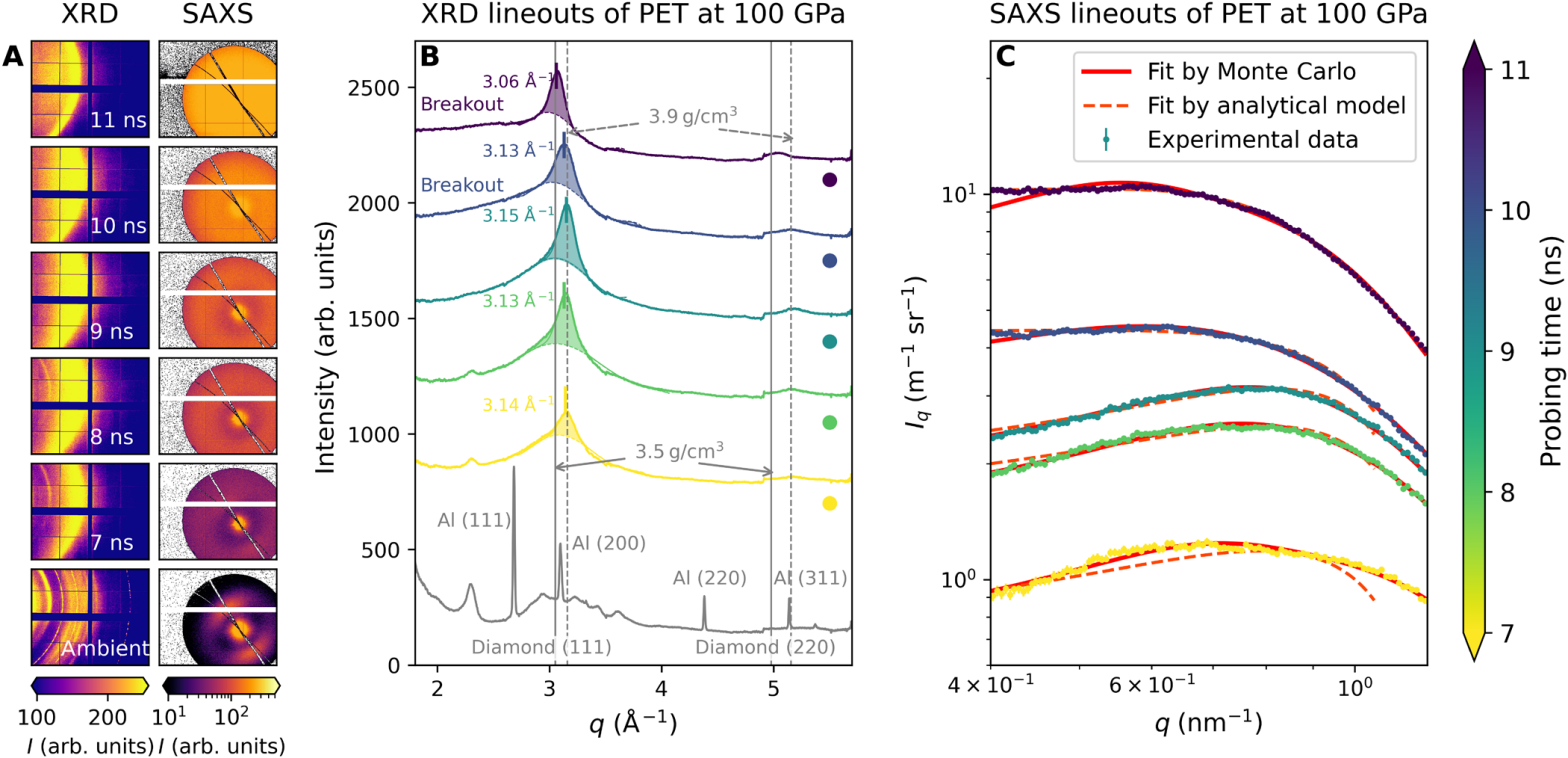
XRD and SAXS results of a 100-μm PET measured at the LCLS with different time delays. (**A**) Raw XRD and SAXS images and corresponding azimuthally integrated lineouts of (**B**) XRD and (**C**) SAXS with increasing pump-probe delay time at around 100 GPa (with laser energies of ~36 J). Offsets of the intensities are applied to different probing time in XRD indicated by the filled colored dots. The dominant error in SAXS lineouts is the Poisson error (*58*). The fit curves from two SAXS analysis methods (see Materials and Methods for details) are plotted.

Figure 2 depicts the temporal evolution of 100-μm PET samples shock-compressed to ~100 GPa at the LCLS in the form of raw in situ XRD and in situ SAXS data with their corresponding azimuthally integrated lineouts. The individual pressures were determined by measuring the shock velocity and using the known PET Hugoniot curve (*15*). In each XRD lineout, the diamond (111) peak illustrated by the shaded area was modeled to be approximately Lorentzian, while the signal below is given by the contribution of a liquid C─H─O mixture, which is well represented by a Gaussian in the *k* range below the diamond peak (*14*). The Gaussian shape is also in good agreement with DFT-MD simulations of the liquid (see Materials and Methods and Supplementary Materials for more details). Due to the experimental geometry, the signal-to-background ratio of the diamond (220) peak is substantially lower than for the (111) reflection. As the (220) peak also sits on top of a liquid correlation peak, the solid contribution is difficult to distinguish quantitatively from the background. Thus, the analysis of the formation characteristics of the nanodiamonds mainly focuses on the more prominent (111) peak. We use the Lorentzian fit to obtain the center and the width of the diamond (111) peak. The Scherrer formula (*22*) was then applied to estimate the minimum crystallite size of a few nanometers based on the width of the diamond Bragg peak of around 0.2 Å^−1^. This value is considerably smaller than the peak width of the liquid mixture peak of 1–2 Å^−1^ by Lütgert *et al.* (*15*) and estimations from DFT-MD simulations. With this clear feature separation, the diamond fraction (the absolute amount of carbon atoms in the shock-compressed PET foil that ended up in diamond lattices) was estimated from the diamond peak integral between the liquid peak and the experimental data (*14*). The diamond diffraction peak area increases with proceeding time during the compression process before shock breakout at the sample rear side (it refers to the cases when the probing times are at 7, 8, and 9 ns since the breakout time at ~100 GPa is about 9.8 ns). Compressed diamond densities of up to 3.87 g/cm^3^ were deduced from the position of the diamond Bragg peak at 3.14 ± 0.01 Å^−1^ compared to the ambient diamond density of 3.51 g/cm^3^ at 3.05 Å^−1^. The diamond peak area stops increasing after shock breakout, and the peak position moves back to lower *q* (~3.06 Å^−1^) as the crystallites are relaxing to ambient density. In SAXS, a substantial increase in total scattering intensity for the progressing shock wave is observed when diamond formation can be seen in XRD data. The described SAXS ring feature is not visible for drives where no diamond formation has been observed. Therefore, the signal is assumed to be generated by the nanodiamonds where both the relative volume fraction and the size distribution of the crystallites affect the overall shape and intensity of the SAXS signal. SAXS is also sensitive to demixing processes without the formation of crystalline structures, as long as density differences are present between the separated states. Therefore, we can conclude that at pressures below and above the conditions where diamond formation is observed, our sample remains in a mixed state.

**Fig. 3.**
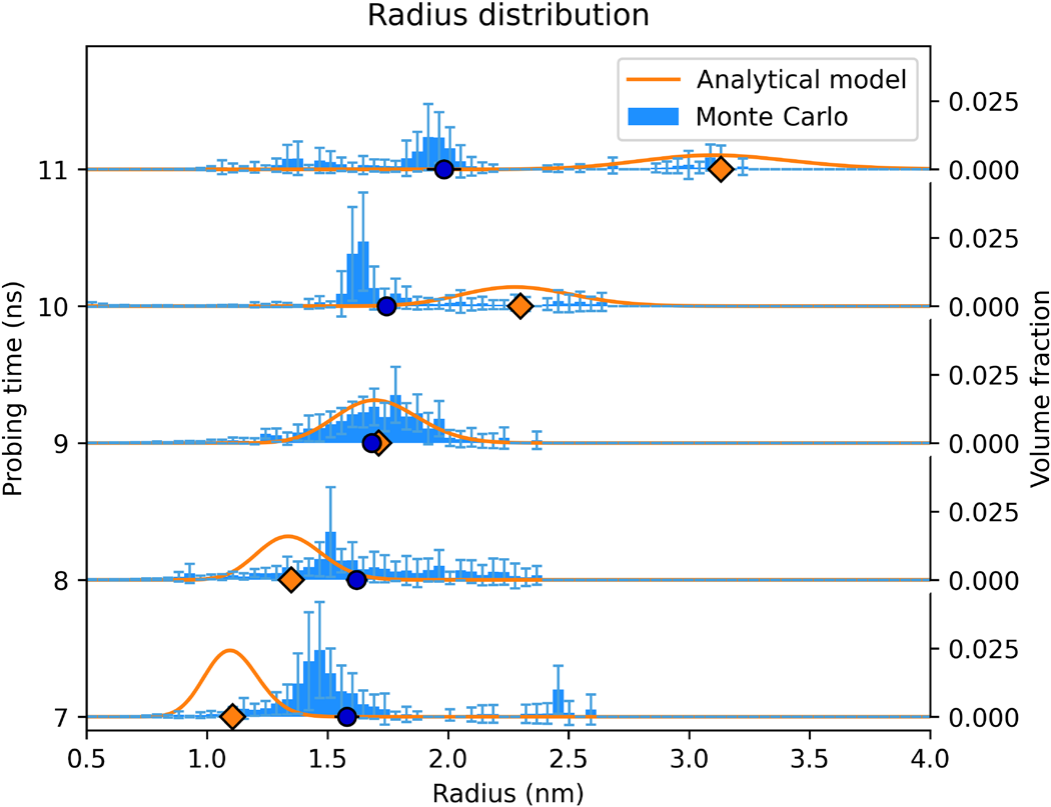
Radius distribution of nanoparticles with compression history. The radius distribution was extracted from the Monte Carlo method and the analytical model corresponding to the SAXS lineouts in Fig. 2C. Dark blue dots and orange diamonds represent the mean radius of the Monte Carlo method and the effective radius of the analytical model, respectively. The uncertainty in the mean radius of the Monte Carlo method comes from the SDs for the individual bars, describing the convergence of the Monte Carlo method.

The high-quality SAXS signals obtained by the detector allow the nanoparticle size distribution to be deduced. Here, we use two different methods for SAXS data analysis (see Materials and Methods and Supplementary Materials for details on the applied methods). Figure 3 shows the obtained nanoparticle distribution derived from the SAXS lineouts in Fig. 2C. As an analytical model, we assumed a Schulz distribution (*23*, *24*) with the polydispersity *p* = σ/*R* ≈ 0.1, where the effective radius *R* was obtained by fitting the shape of the SAXS lineouts, and σ was the root mean square deviation from *R*. Because of the high data quality and a sufficient *q* range, the radius distribution of the nanodiamonds can also be obtained by Monte Carlo methods without assuming a specific distribution function. Here, the mean radius of particles increases from 1.6 to 2 nm with increasing delay time between the drive laser and x-ray probe. After the shock breakout, which results in a pressure release of the partially solid and liquid system, the Monte Carlo results indicate that the radius distribution becomes highly dispersed (see Fig. 3). At this stage, the analytical model results in systematically larger effective radii. However, the simple assumption of a Schulz distribution for moderately dispersed systems seems no longer valid at this point. In summary, the growth of the mean particle size with progressing shock propagation has been observed by both methods, indicating that the nanodiamonds increase their size until the shock breakout releases the high-pressure conditions.

### Effect of pressure

PET samples were driven to different shock pressures by varying the pulse energy of the drive laser to investigate the effect of pressure and temperature on the diamond formation in shock-compressed C─H─O samples. At pressures below 74 GPa, both XRD and SAXS data did not show diamond formation. For intermediate pressures between 74 and 125 GPa, diamond features are present in the data. However, they disappear above 125 GPa. At this upper bound in pressure, temperatures approach the diamond melting line (*25*), reaching approximately 6000 K along the PET Hugoniot (*15*). Results from the different facilities, LCLS and SACLA, provide excellent agreement on this observation, which reduces a potential effect of systematic errors in the individual experimental setups.

Using DFT-MD simulations, we can compare our experimental XRD lineouts with calculated diffraction patterns of the compressed C─H─O mixtures with various carbon-to-water ratios at different pressures along the PET Hugoniot. The simulated patterns are reasonably consistent with the residual liquid structure and inferred temperatures from our experimental data (see Materials and Methods and Supplementary Materials for more details).

It is observed that the SAXS intensity during shock propagation is directly correlated to the diamond content recorded by XRD (see fig. S3 for details). Moreover, data from both SAXS analysis methods consistently showed evidence that the nanodiamond particle sizes grow with increasing pressure (see fig. S4 for details). One reasonable explanation is that high pressure promotes and accelerates the growth of the particle size, resulting in a larger size at higher pressure as probed by x-ray techniques during the propagation of shock waves with various intensities through samples of the same thickness.

## DISCUSSION

Using XRD, we were able to confirm the diamond formation from compressed PET in laser-driven shock experiments. In SAXS data, a prominent signal was observed that we identified with nanoparticles with several nanometers in diameter. Since this feature occurred when a strong diamond signal was detected in in situ XRD data, we infer that the nanoparticles observed in SAXS are likely to be diamond crystals. Therefore, SAXS provides a sensitive instrument to study diamond formation in shock-compressed experiments, even when the signal of nanoparticles is weak and XRD is impeded by the liquid background peak. These findings were reproduced over different target materials and different setups at different international laser facilities (LCLS and SACLA).

**Fig. 4.**
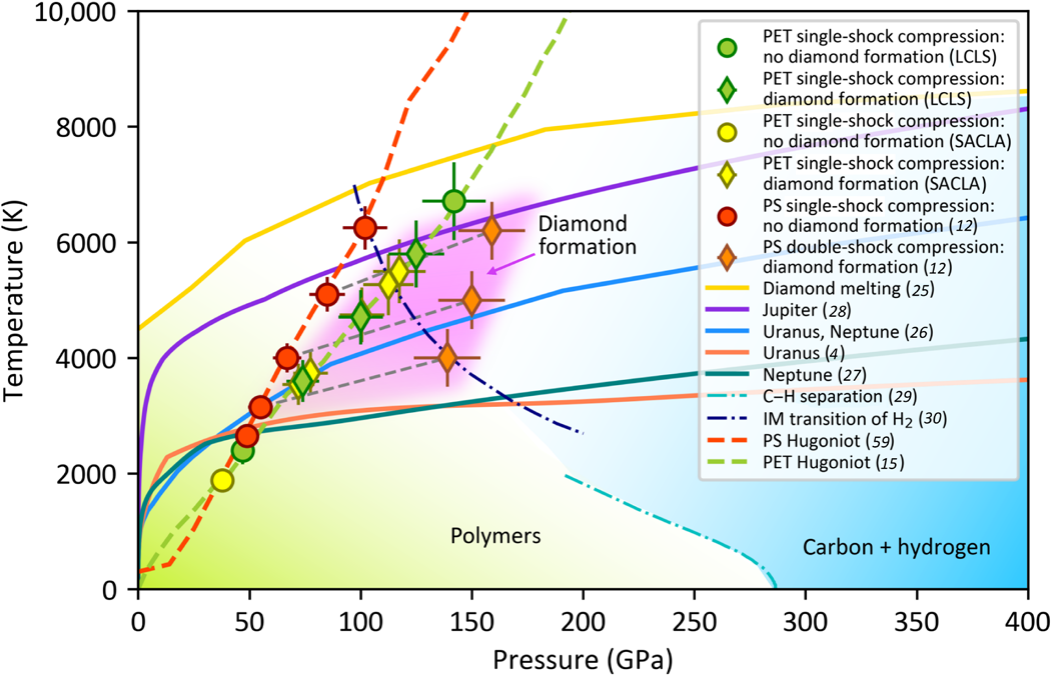
Phase diagram of mixtures of carbon, hydrogen, and oxygen. The predicted isentropes of Uranus (*4*, *26*), Neptune (*26*, *27*), and Jupiter (*28*); the diamond melting line (*25*); the C─H separation line (*29*); the insulator-metal (IM) transition curve (*30*); the Hugoniot of PS (*59*) and PET (*15*); experimental data of PS in previous works (*12*, *13*); and PET in this work are all presented. The error bars are obtained from the comparison of the XRD measurement with predictions of the scattering intensity from DFT-MD simulations (see Materials and Methods).

In Fig. 4, our results for shock-compressed PET are illustrated in a phase diagram together with previous data from PS (*12*), models for planetary interiors (*4*, *26*–*28*), theoretical predictions for diamond melting (*25*), and C─H phase separation (*29*) as well as the assumed hydrogen insulator-metal transition (*30*). The data points where we observe diamond formation in plastics are marked by diamond symbols in the purple shaded area. This area overlaps with the predicted isentropes of Uranus and Neptune (*26*) (represented by only one curve because of the small differences), but is a little bit higher in temperature than the more recent models (*4*, *27*), and slightly intersects the recently predicted isentrope for Jupiter (*28*). In PET, diamond forms under *P*-*T* conditions where no C─H separation has been observed for PS on the principal Hugoniot. As the temperature on the PET Hugoniot is only marginally smaller, especially at lower pressures, this suggests that the oxygen atoms in PET are key for explaining the difference, in agreement with first-principles studies of mixtures relevant to the interiors of ice giants (*3*). In the first-principles studies, it was found that the presence of O increases the lifetimes of C─C bonds, resulting in the clustering of C atoms and the demixing of the liquid mixtures in such environments (*3*).

Moreover, our results of *P*-*T* conditions for diamond formation roughly coincide with the insulator-metal transition of liquid hydrogen suggested by theoretical (*30*) and experimental studies (*31*–*35*), which may indicate a direct correlation. The differences between the PS and PET results, however, point toward a substantial influence of the initial composition on chemistry under extreme conditions. In this case, our study demonstrates that the presence of oxygen supports diamond formation, which is therefore likely to occur inside planets of the corresponding composition. If carbon would be oxidized, diamond formation would have been reduced in our experiment in comparison to the pure C─H data. As the opposite is observed, it can be concluded that oxygen supports rather than inhibits C─H separation. The subsequent carbon precipitation can then act as a heat source inside ice giants, leading to a carbon-rich layer around the core. Carbon separating from H and O also increases the likelihood for regions of superionic water and/or metallic hydrogen being present inside the ice giants (*3*, *36*), which may help to explain the peculiar magnetic fields observed for those planets.

Our study has shown that simultaneous use of in situ x-ray methods in dynamic compression experiments at XFEL facilities provides insight of unprecedented quality into the peculiar chemistry deep inside planets to obtain a better understanding of our Solar System and the vast number of exoplanets found in the orbit of other stars. In particular, the demonstrated experimental platform can be used to investigate various mixtures of light elements to obtain a most complete picture of the chemical processes in the ice giants. Shock drivers with higher repetition rates will enable studies of many different stoichiometries and better access to kinetics, e.g., via detailed scans of P/T conditions and varying the sample thickness.

Last, the observation of nanodiamonds with tailored sizes in the range of a few nanometers may open the path toward an efficient new source of this material, which exhibits a steadily increasing number of applications in science, medicine, catalysis, and electronics (*37*). In particular, nanodiamonds of these small sizes with specific color centers may require more effective synthesis methods than currently available to satisfy the needs of applications (*38*). State-of-the-art high-energy laser systems with hertz repetition rates together with adequate recovery methods (*39*) may be able to produce large amounts of specifically tailored nanodiamonds from cheap PET. As demonstrated here, this initial material provides access to the diamond formation regime by single-shock compression and therefore does not require sophisticated laser pulse–shaping techniques. Nanodiamonds with specific color centers may be achieved by the corresponding dopant of the initial PET plastics.

## MATERIALS AND METHODS

### Experimental details

During experiments at LCLS (see similar setup on SACLA in fig. S1 of the Supplementary Materials), a drive laser shock-compressed 100-μm-thick PET foils with a flat top pulse profile, fluences of 15 to 62 J in 8 ns (full width at half maximum), and a spot size of 300 μm in diameter, resulting in intensities between 2.7 and 11 TW/cm^2^. The drive laser creates a rapidly expanding plasma on the front surface of the target, launching a single shock wave into the cold material behind the ablation front. In contrast to the previous experiments on PS (C_8_H_8_)*_n_* using a double-stage shock (*12*, *13*), a single pulse is sufficient to drive C─H─O samples to similar planetary-relevant states (*15*). An aluminum coating with a thickness of 1 μm was coated on the sample to prevent low-intensity laser prepulses to preheat the sample, and the reflective metal layer can also be applied to characterize the shock dynamics with VISAR to constrain pressure and density inside the sample. In front of the aluminum coating was a 9-μm-thick PS ablator.

XRD was performed in an angular range from 2θ = 22° to 72° (corresponding to scattering vector lengths from *q* = 1.8 to 5.6 Å^−1^) with ePix detectors (*40*). For SAXS, a Jungfrau detector (*41*) located 1165 mm from the target allowed to measure an angular range from 2θ = 0.24° to 1.31° (corresponding to scattering vector lengths from *q* = 0.2 to 1.1 nm^−1^).

Assuming a stable shock wave transmitting in the sample, the average shock velocity in the compressed sample is given by the transit time via VISAR, as shown in Fig. 1. The pressure and temperature in the sample are then derived from the Hugoniot of PET (*15*). Those results were cross-checked with the *P*-*T* conditions by comparison of the XRD data with calculations from DFT-MD simulations. Both approaches agreed within relative errors of ±10% in temperature and pressure.

### SAXS analysis methods

Two SAXS data analysis methods are applied in this work to obtain the size distribution of nanoparticles. The classical SAXS analysis is based on an analytical model. The expected scattering intensity *I*_abs_ in absolute units can be defined using (*21*)

$$I_{\text{abs}}(q)=\varphi (1-\varphi )\cdot {\left(\Delta \rho_{\text{sl}}\right)}^{2}\cdot V\cdot {|F(q)|}^{2}\cdot S(q)$$ (1)

where *q* is the wave number, φ is the volume fraction of the nanoparticles, 1 − φ is the volume fraction of the solution, *V* is the volume of a single nanoparticle, Δρ_sl_ is the total scattering length density contrast between solids and solutions, and *F*(*q*) and *S*(*q*) represent the form factor and structure factor, respectively. Considering that the distribution of nanoparticles in the experiments is polydisperse in size and the intensity can be regarded as the average of all size contributions, the form factor *F*(*q*) of spherical nanoparticles with a Schulz distribution (*23*, *24*) assuming that the diamonds exist in a uniform liquid background can be expressed as

$${|F(q)|}^{2}=\frac{\pi^{2}}{2}\cdot {\left(z+1\right)}^{-6}\cdot a^{z+7}\cdot G(q)$$ (2)

where

$$\begin{array}{c} a=\frac{z+1}{q\cdot R}\\ G(q)=a^{-(z+1)}-{\left(4+a^{2}\right)}^{-\frac{z+1}{2}}\cdot \text{cos}((z+1)\cdot \text{arctan}(\frac{2}{a}))+\\ (z+1)\cdot (z+2)\cdot (a^{-(z+3)}+{\left(4+a^{2}\right)}^{-\frac{z+3}{2}}\cdot \text{cos}((z+3)\cdot \text{arctan}(\frac{2}{a})))-\\ 2\cdot (z+1)\cdot {\left(4+a^{2}\right)}^{-\frac{z+2}{2}}\cdot \text{sin}((z+2)\cdot \text{arctan}(\frac{2}{a})) \end{array}$$ (3)

Also, *z* = 1/*p*^2^ − 1, *p* = σ/*R* describes the polydispersity of the nanoparticles. By introducing the repellent potential *U*(*r*) of a hard sphere in the Percus-Yevick closure (*42*–*45*), the structure factor can be described as

$$S(q)=\frac{1}{1+24\cdot \varphi \cdot \frac{G(2\mathrm{qR})}{2\mathrm{qR}}}$$ (4)

where

$$\begin{array}{c} a=\frac{{\left(1+2\varphi \right)}^{2}}{{\left(1-\varphi \right)}^{4}}\\ \beta =-6\varphi \cdot \frac{{\left(1+\frac{\varphi }{2}\right)}^{2}}{{\left(1-\varphi \right)}^{4}},\\ \gamma =\frac{1}{2}\varphi \cdot \frac{{\left(1+2\varphi \right)}^{2}}{{\left(1-\varphi \right)}^{4}},\\ G(A)=\frac{a}{A^{2}}\cdot (\text{sin}(A)-A\cdot \text{cos}(A))+\\ \frac{\beta }{A^{3}}\cdot (2A\cdot \text{sin}(A)+(2-A^{2})\cdot \text{cos}(A)-2)+\\ \frac{\gamma }{A^{5}}\cdot (-A^{4}\cdot \text{cos}(A)+4\cdot [(3A^{2}-6)\cdot \text{cos}(A)+(A^{3}-6A)\cdot \text{sin}(A)+6]) \end{array}$$ (5)

Therefore, the particle distribution and an associated goodness of fit can be obtained by fitting this analytical model to the experimental data.

In addition, SAXS data can be analyzed using a Monte Carlo technique to fit the experimental data, which is very suitable for complex structures. It is even possible to fit points of an arbitrary radial distribution assuming densely packed spheres with the SAXS analysis program McSAS (*46*, *47*) in the premise of high-quality data with known error and absolute units. It should be considered that each sampling point within the radial distribution function adds another free parameter in the fit, so it is more suitable for data with a wide *q* range and high dynamic range. Also, this distribution best applies to systems with a single scattering component as in our case (only one type of polydispersed nanoparticles).

### DFT-MD simulations

The DFT-MD simulations of diffraction signals in compressed C─H─O samples with various carbon-to-water ratios under different pressure-temperature conditions were compared to experimental XRD data, which can help to predict the demixing of nanodiamonds and residual liquid in the sample. The simulations were performed using the VASP package (*48*–*51*), where the Mermin formulation was used to optimize the Helmholtz free energy at a given temperature (*52*). The electronic density in the simulation box with periodic boundary conditions was represented by a plane wave expansion with a cutoff energy of *E*_cut_ = 1000 eV. The electron-ion interaction was modeled using the projector augmented wave (PAW) approach, specifically the hard PAW pseudopotentials for hydrogen (H_h, 06Feb2004), carbon (four valence electrons, C_h Feb2004), and oxygen (six valence electrons, O_h Feb2004) as provided with VASP (*53*, *54*). The exchange-correlation functional was taken in generalized gradient approximation in Perdew-Burke-Ernzerhof parametrization (*55*). The Brillouin zone of the supercell was generally sampled at the Γ point only. The electronic bands were populated using a Fermi distribution at the chosen temperature. The supercell contained atoms of carbon, hydrogen, and oxygen (ratio of C to H_2_O was set from 0.31:1 to 2.5:1 where 2.5:1 is the C-to-H_2_O ratio of cold PET, and there are always 32 units of H_2_O in the supercell), whose movements were calculated using the Hellmann-Feynman forces derived from the electron densities of DFT under the Born-Oppenheimer approximation. The time step was *t* = 0.2 fs, and the DFT-MD run covers a time span of 2 to 4 ps. The ion temperature was controlled by a Nosé-Hoover thermostat (*56*). The ion structure and therefore the intensity of the elastic x-ray scattering in this multicomponent system can be obtained from the recorded MD coordinates (*57*). The expected x-ray scattering intensity *I* was calculated using

$$I(q)\propto \sum_{\mathrm{ab}}\sqrt{x_{a}x_{b}}f_{a}(q)f_{b}(q)S_{\mathrm{ab}}(q)+\sum_{a}x_{a}\sum_{n}(1-f_{\text{an}}{\left(q\right)}^{2})$$ (6)

where *x_a_* is the atomic fraction of species *a* (with *a* being O, C, or H in various C─H─O samples), *f_a_* is the atomic form factor of the whole ion or atom *a*, and *f_an_* is the contribution to *f_a_* caused by the *n*th electron. *S_ab_* denotes the partial structure factors. The first item to the right of proportional sign in Eq. 6 describing the elastic scattering is determined by the Rayleigh weight (*54*). The second term considers the inelastic scattering as it was treated by Lütgert *et al.* (*15*).

## Supplementary Materials

### This PDF file includes:

Table S1 Sections S1 to S4 Figs. S1 to S5
