# Supplementary material for: Diamond formation kinetics in shock-compressed C─H─O samples recorded by small-angle x-ray scattering and x-ray diffraction
Source: Sci Adv. 2022 Sep 2;8(35):eabo0617. doi: 10.1126/sciadv.abo0617 (PMC10848955; doi:10.1126/sciadv.abo0617)
Supplement: Supplementary file 1 — Table S1 Sections S1 to S4 Figs. S1 to S5 [file sciadv.abo0617_sm.pdf]

Science Advances
AAAS

Supplementary Materials for

Diamond formation kinetics in shock-compressed C─H─O samples recorded by small-angle x-ray scattering and x-ray diffraction

Zhiyu He *et al.*

Corresponding author: Zhiyu He, zhiyu.he@uni-rostock.de; Dominik Kraus, dominik.kraus@uni-rostock.de

Sci. Adv. **8**, eabo0617 (2022)
DOI: 10.1126/sciadv.abo0617

This PDF file includes:

Table S1
Sections S1 to S4
Figs. S1 to S5

Supplementary Materials

Table S1. Experimental data. The corresponding *P-T* relation and the effect of pressure on diamond fraction of shocked PET are plotted in Fig. 4 and Fig. S4, respectively.

XFEL	Run	Thickness/ μm	Delay /ns	Pressure/ GPa	Temperature/ K	Diamond fraction/ %	Time after breakout/ns
LCLS	188	100	7	100±10	4700±470	10.0	-2.80
	186	100	8	100±10	4700±470	13.0	-1.92
	182	100	9	100±10	4700±470	15.0	-0.98
	190	100	10	100±10	4700±470	9.0	0.002*
	192	100	11	100±10	4700±470	7.2	2.25*
	282	100	7	142±14	6700±670	0	-1.47
	286	100	8	125±13	5800±580	1.5	-1.02
	289	100	10	74±7	3600±360	18.0	-1.28
	298	100	13	47±5	2400±240	0	-0.78
SACLA	45	100	8	117±12	5498±550	0.6	-0.34
	96	50	5.5	101±10	4745±475	7.8	0.99*
	50	50	7	72±7	3543±354	7.4	1.82*
	55	50	5	113±11	5268±527	3.0	0.71*
	100	50	5.5	78±8	3736±374	12.6	0.48*
	97	50	5.5	38±4	1884±188	0	-1.21

* Breakout runs

Section S1. Experimental details on SACLA

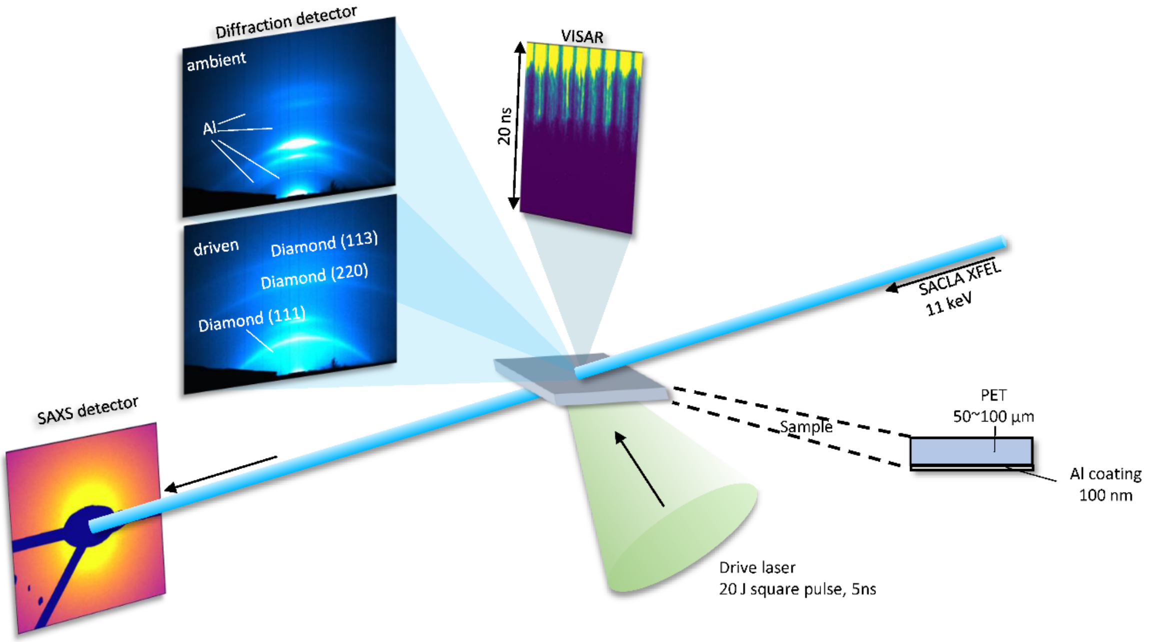

Fig. S1.

Schematic of the experimental setup combining XRD, SAXS and VISAR in one experiment at SACLA. A drive laser shock-compressed 50 or 100 µm thick PET foils with a flat-top pulse profile and fluences of 5-22 J in 5 ns (full width at half maximum), and a spot size of 200 µm in diameter resulting in intensities between 2.4-10.5 TW/cm^2. An aluminum front layer with a thickness of 100 nm was coated on the sample to prevent low intensity laser pre-pulses from preheating the sample. The reflective metal layer can also be applied to characterize the shock dynamics with VISAR to constrain pressure and density inside the sample. The structural changes and density variations of compressed samples can be observed by *in situ* XRD and SAXS with the SACLA pulse of 11.1 keV photon energy. XRD was performed in an angular range from $2\theta = 22.6°$ to 77° (corresponding to scattering vector lengths from q = 2.2 Å^{-1} to 7 Å^{-1}) with XRD detectors. For SAXS, the detector was located 1180 mm from the target, providing an angular range from 2θ = 0.16° to 1.3° (corresponding to scattering vector lengths from q= 0.16 nm^{-1} to 1.28 nm^{-1}).

Section S2. Analytical SAXS Model

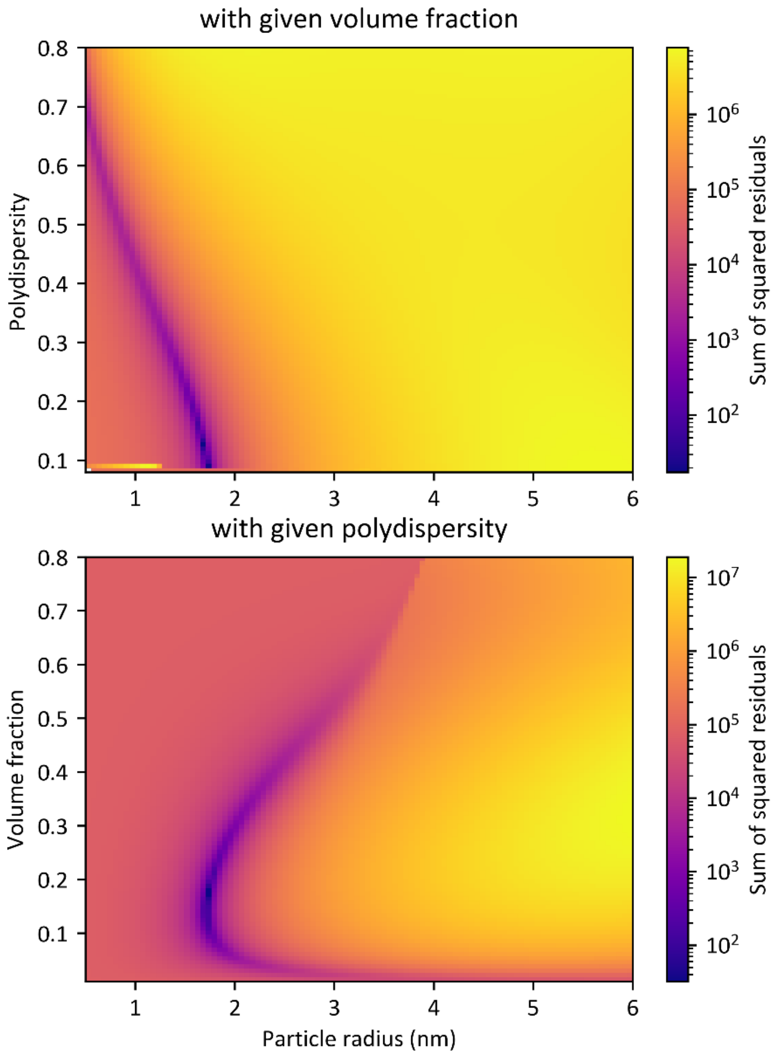

Fig. S2.

Sum of the squared residuals of the analytical model and experimental data (run 182, ~100 GPa, 9 ns, see Fig.2 and Fig.3 in the main text). The top illustration depicts the influence of effective particle size and polydispersity on the optimal analytical model with a given volume fraction of 15 %, indicating that the best polydispersity of the system is around 0.1 (the assumed Schulz distribution is only applicable when the polydispersity is higher than 0.08). The bottom illustration describes the influence of effective particle size and volume fraction on the optimal analytical model with a given polydispersity of 0.1, demonstrating the consistency in volume fraction between the optimal analytical model and that derived from XRD lineout. Both illustrations point out the best effective particle radius (~1.7 nm) under the assumption of the analytical model.

Section S3. Effect of conditions

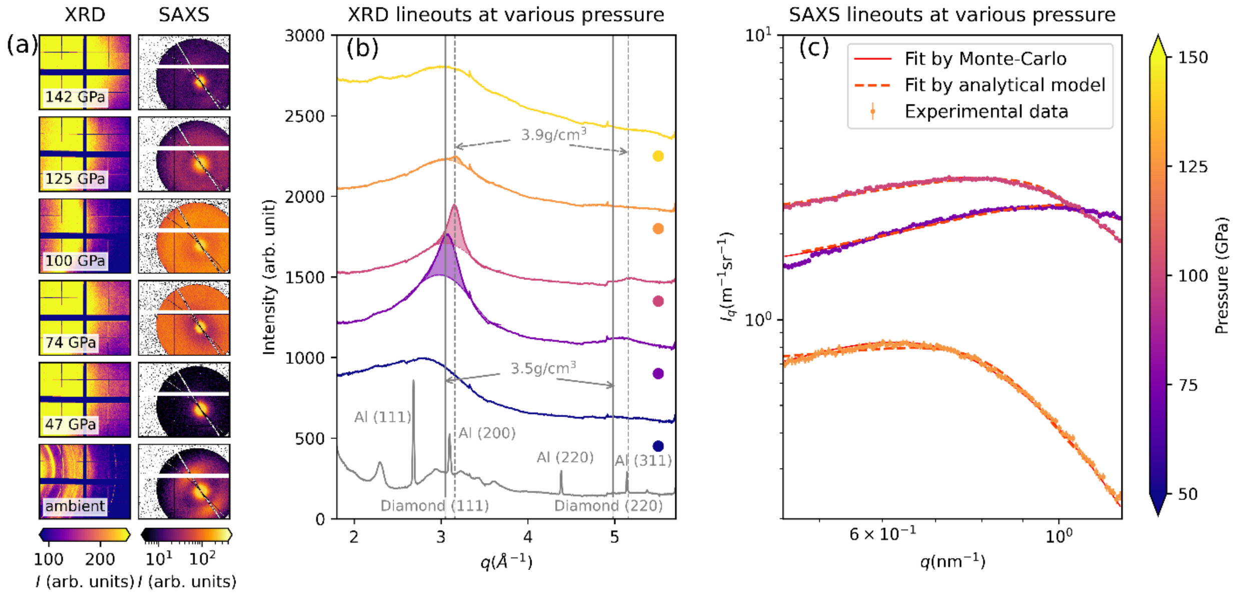

Fig. S3.

(a) Raw XRD and SAXS images of 100 μm PET measured at the LCLS and corresponding azimuthally integrated lineouts of (b) XRD and (c) SAXS at various pressures. The dominant error in SAXS lineouts is the Poisson error. Almost no diamonds form in the low-pressure region (below 50 GPa). The diamond peak appears in the intermediate-pressure region and disappears in the high-pressure region (above 125 GPa). The feature intensity of the SAXS lineout first increases and then decreases with the growing pressure, which is strongly correlated to the diamond content observed by XRD.

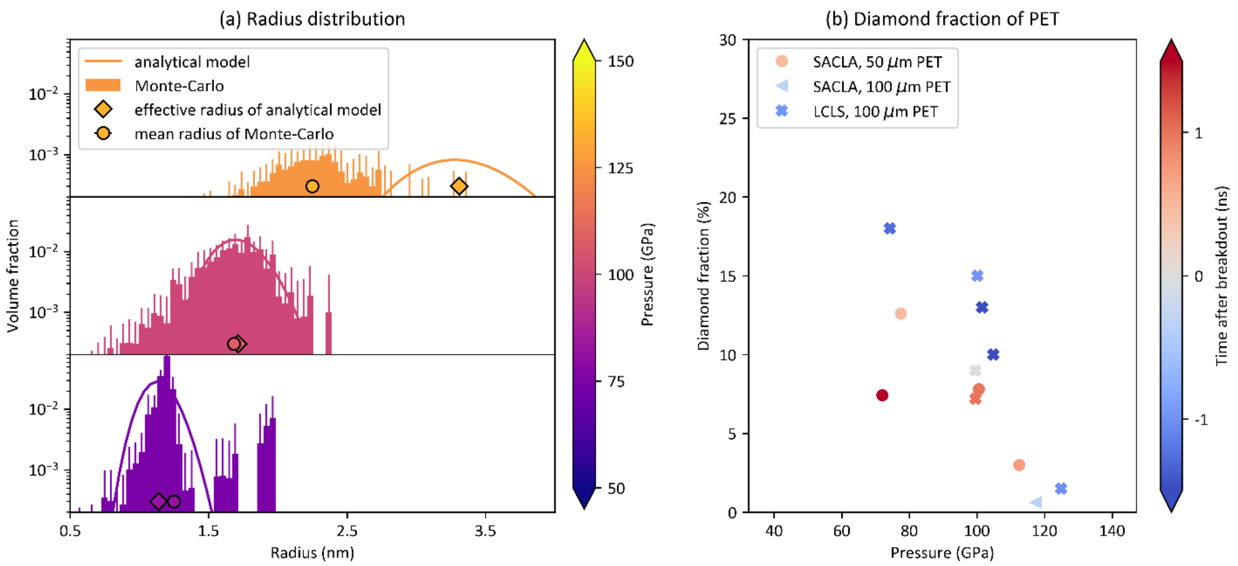

Fig. S4.

(a) Radius distribution of nanoparticles with growing pressure extracted from the Monte-Carlo method and the analytical model corresponding to the SAXS lineouts in Fig. S3(c). Dots and diamonds represent the mean radius of the Monte-Carlo method and the effective radius of the analytical model, respectively. The uncertainty in the mean radius of the Monte-Carlo method comes from the standard deviations for the individual bars, describing the convergence of the Monte-Carlo method. (b) Effect of pressure vs diamond fraction in shock-compressed PET obtained from LCLS and SACLA.

Section S4. DFT-MD simulations

In Fig. S5, *in situ* XRD lineouts of shock-compressed PET at different pressures are compared to DFT-MD simulation data of C-H-O mixtures at different *P*-*T* conditions. The experimental XRD lineout was selected at the time just before breakout, which is the point where the conditions inside the target are most homogeneous. Scattering lineout of simulations for temperatures from 4000 K to 10000 K, pressures from 41 to 274 GPa and C to H_2O ratios x from 0.31:1 to 2.5:1 were evaluated. Squared deviations v^2 of the DFT-MD predictions C_i from the experimental data M_i were calculated to fit the artificial XRD signal to the measurement

$$v^2 = \frac{1}{N-1} \sum_i (M_i - C_i)^2 \tag{S1}$$

where N is the number of scattering vector points. Fig. S4 (a) and (b) describe the XRD lineout of shocked PET at 100 and 125 GPa, respectively, whose pressure is determined by the DFT-MD Hugoniot of PET according to the shock velocity measured by VISAR. The indicator v^2 favours the prediction for T = 6000 K at P = 93 GPa and x = 2.5 (v^2 = 0.24) corresponding to Fig. S5(a) and the prediction for T = 10000 K at P = 115 GPa and x = 2.5 (v^2 = 0.13) corresponding to Fig. S5(b). The higher intensity of experimental data at small diffraction angles might be explained by a small amount of remaining cold material. The increase of pressure from ~100 GPa to ~125 GPa would cause the shift of the liquid peak towards higher q and the decrease of the diamond peak while x has a relatively weak effect on the scattering intensity. The simulated scattering lineout is reasonably consistent with the residual liquid structure in the experimental data and can roughly predict the content of the diamond peak. However, the demixing of carbon and H_2O and diamond formation cannot be directly observed in the simulations.

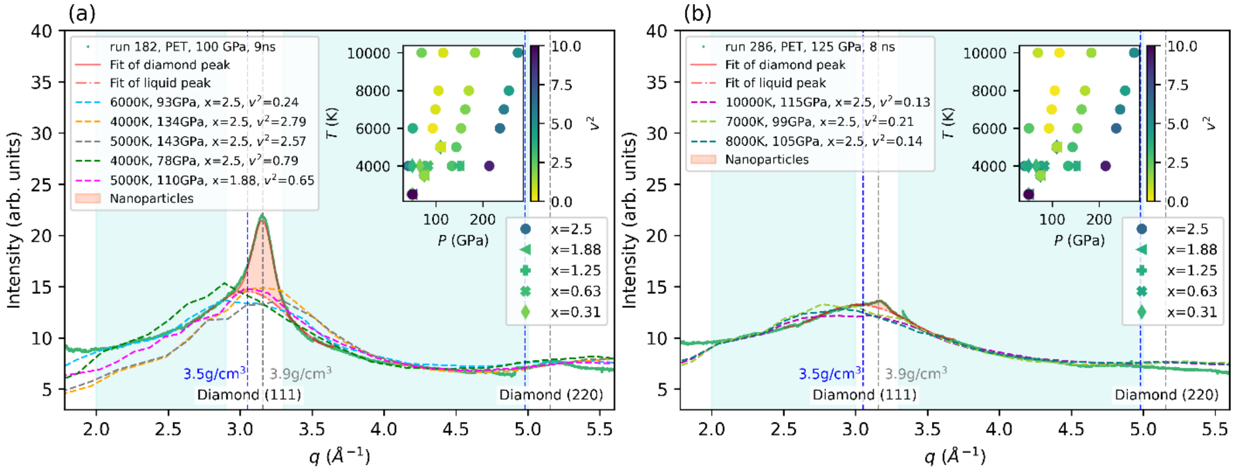

Fig. S5.

Comparison of the experimental diffraction lineout to predictions via DFT-MD calculations of C-H-O mixtures in which x represents the C to H_2O ratio. The q regions marked in light cyan were used to fit the experimental data in the DFT-MD simulations. The squared deviations v^2 (see Eq. S1) are plotted for different simulation parameters in the inset map. The diamond Bragg peak is highlighted by a peach-colored filling in the experimental but not the simulated data.
